# *Inquilinus* Species Infections in Humans—A Narrative Review

**DOI:** 10.3390/microorganisms13030592

**Published:** 2025-03-04

**Authors:** Anastasia Vasilopoulou, Takis Panayiotou, Stella Baliou, Andreas G. Tsantes, Petros Ioannou

**Affiliations:** 1 School of Medicine, University of Crete, 71003 Heraklion, Greece; 2 Laboratory of Hematology and Blood Bank Unit, “Attikon” University Hospital, School of Medicine, National and Kapodistrian University of Athens, 12462 Athens, Greece

**Keywords:** *Inquilinus*, cystic fibrosis, infection, infective endocarditis

## Abstract

Background: *Inquilinus* species are Gram-negative, non-sporulating, non-pigmented rods that are catalase-positive, indole-negative, and able to grow at various temperatures and in 1% NaCl. Infections due to *Inquilinus* spp. are increasingly identified, especially in patients with cystic fibrosis. Objective: This review aims to present all reported cases of *Inquilinus* spp. infections in humans, with an emphasis on data regarding epidemiology, antimicrobial resistance, antimicrobial treatment, and mortality. Methods: A narrative review based on a literature search of the PubMed/MedLine and Scopus databases was performed. Results: In total, 13 articles providing data on 25 patients with *Inquilinus* infections were included in the analysis. The median age was 19 years, while 60% were male. Cystic fibrosis was the predominant risk factor (92%). Respiratory tract infection was the most common type of infection (96%). *Inquilinus limosus* was the most commonly identified species. Polymicrobial infection was very common (77.3%). Microbial identification required the use of advanced molecular techniques, such as 16s rRNA sequencing. The pathogen exhibited resistance to beta-lactams, with the exception of carbapenems. The most commonly used antimicrobials included carbapenems (68.4%), followed by quinolones (57.9%) and aminoglycosides (52.6%). Mortality was low (4%). Conclusions: Due to the potential of *Inquilinus* spp. to cause infection in patients with cystic fibrosis, and given the difficulties in microbial identification, clinicians and laboratory professionals should consider it in the differential diagnosis of patients with cystic fibrosis and respiratory tract infection not responding to beta-lactam treatment or with polymicrobial infections, especially when traditional techniques are used for microbial identification.

## 1. Introduction

Cystic fibrosis is a genetic, autosomal recessive disease caused by variants of the *CFTR* gene that manifests most commonly during childhood. *CFTR* encodes for an ion channel, CFTR, which regulates the balance of water and electrolytes on the surface of several tissues, such as the biliary tract, the pancreas, the cervix, the vas deferens, the intestine, the upper and lower airways, and the sweat glands [1,2]. These variants are associated with fluid imbalance in the lungs and other organs. This fluid imbalance in the lungs is associated with the production of thickened secretions, reduced transport through the mucociliary system, and mucus retention. This retention can lead to reduced bacterial cleaning, leading to the persistence of bacteria that may then increase the likelihood of infections [1,2,3,4]. There is a strong association of cystic fibrosis with specific microorganisms either in the context of colonization or in that of infection. Such microorganisms are *Staphylococcus aureus*, *Pseudomonas aeruginosa*, *Achromobacter* spp., *Burkholderia cepacia* complex, and *Stenotrophomonas maltophilia* [1,2]. New species with pathogenic potential are currently being discovered with the use of new molecular identification methods in microbiology. Such previously unknown species include *Pandorea apista, Ralstonia pikettii*, and *Inquinus limosus* [5]. Such species, such as *Inquilinus* spp., have been identified in patients with cystic fibrosis both as colonizers and as pathogens associated with respiratory tract infections [6].

*Inquilinus* species were first identified in 1999 from Pitulle et al. from a clinical specimen of a patient with lung transplantation and cystic fibrosis [6]. Based on a phylogenetic analysis of the small subunit rRNA and biochemical assays, these species were found to be unrelated to other known bacteria up to that time and represented a new genus within the subdivision of α-proteobacteria [6]. Soon after, Coenye et al. identified eight more isolates from the same genus from patients with cystic fibrosis and proposed the novel genus and species of *Inquilinus limosus* based on a whole-cell protein analysis, DNA–DNA hybridization, and 16S ribosomal DNA sequencing [7]. *Inquilinus* spp. are Gram-negative, non-sporulating, non-pigmented rods that are catalase-positive, indole-negative, and able to grow at various temperatures and in 1% NaCl [7]. *I. limosus* has a size of about 1.5 to 2 μm and a length of 3.5 μm [6,7]. They grow best between 35 °C and 42 °C but can also grow at 25 °C. *I. limosus* is not motile, and it can utilize many carbohydrate sources, including L-fucose, D-fructose, maltose, D-galactose, D-mannose, D-sorbitol, and D-raffinose, but not D-glucose [6,7]. Thus, it belongs to the non-fermenting bacteria [8]. Additionally, it cannot utilize sorbitol, mannitol, rhamnose, inositol, melibiose, sucrose, amygdalin, citrate, or arabinose [7]. Among the four DNA sequences of *I. limosus* deposited in GeneBank (DSM 16000, Inq, MP06, and YM_69_17), the total sequence has a length of 5.93–7.69 Mb, a GC content of 69.6–66.9%, and 5877 to 7098 encoding genes [8].

Based on the first studies of *Inquilinus* spp., a close association with cystic fibrosis was noted, even though whether this identification represents colonization or infection may not always be clear and the potential of this microorganism to cause other infections is not clear [6,7].

The aim of the present study was to comprehensively review all the available information of all types of infections caused by *Inquilinus* spp. in the literature and to assess clinical data, microbiology, treatment, and outcomes.

## 2. Materials and Methods

### 2.1. Search Strategy and Inclusion and Exclusion Criteria

This review aims to collect and present all published data on *Inquilinus* species infections in humans. The primary aim was to present data on the epidemiology and mortality of the patients. Secondary aims were to gather data about specific infection sites, clinical presentation, microbiological features, and treatment provided. For this review, the PubMed/Medline and Scopus databases were searched up to 13 December 2024. Data were extracted using a predefined template. The following keywords were applied for the search strategy: “*Inquilinus*” AND “infection”. The inclusion criteria encompassed studies presenting original data, such as case series, case reports, and cohort studies providing information on the epidemiology and clinical outcomes of *Inquilinus* spp. infections in humans. Only studies in the English language were considered. Reviews and systematic reviews were excluded. Studies involving animals, articles without full-text access, and those lacking information on patients’ mortality and epidemiology were excluded from the analysis. Finally, cases of colonization by *Inquilinus* species were excluded from the analysis, based on the clinical course depicted and the study authors’ consideration whether the species were pathogens or colonizers. The references of all included articles were examined to identify any studies potentially missed in the initial search.

### 2.2. Data Extraction and Definitions

The data extracted from each included study were the publication year, article type, country of origin, patient demographics (age and gender), relevant medical history, details of infection, and key clinical characteristics, such as the specific infection site, complications, as well as microbiological characteristics, such as the identified pathogen, antibiotic susceptibilities, and finally, the treatment used and outcome (survival or mortality). The relationship between mortality and the initial infection was documented according to each study’s authors.

### 2.3. Statistical Analysis

Data are presented as numbers (%) for categorical variables and median (interquartile range, IQR) for continuous variables. Continuous variables were compared using the Mann–Whitney U-test for non-normally distributed variables or the *t*-test for normally distributed variables. All tests were two-tailed, and a *p*-value equal to or lower than 0.05 was considered significant. Statistics were calculated with GraphPad Prism 6.0 (GraphPad Software, Inc., San Diego, CA, USA).

## 3. Results

### 3.1. Included Studies’ Characteristics

**Figure 1:**
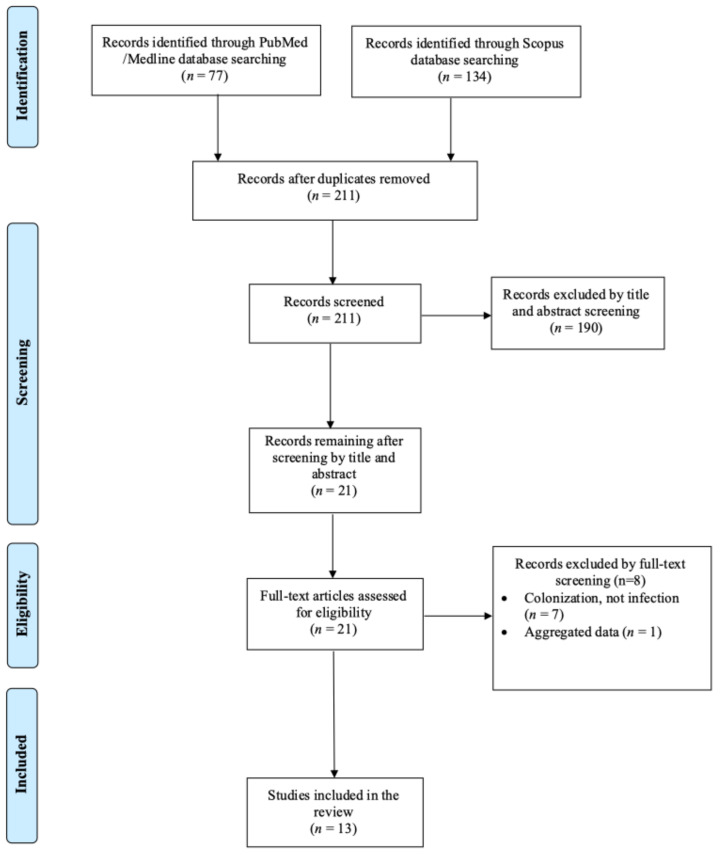
Trial flow of this narrative review.

**Figure 2:**
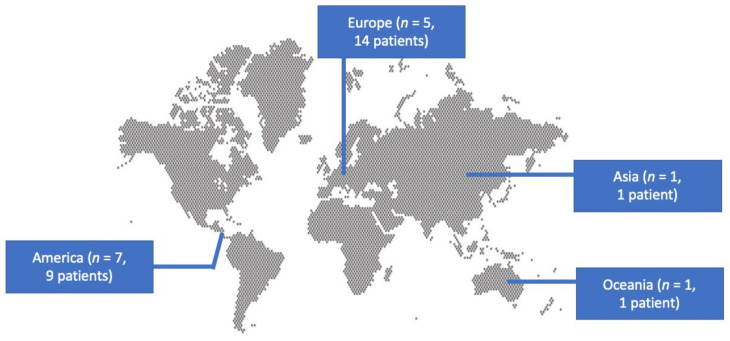
Geographical distribution of *Inquilinus* spp. studies and infections globally.

A total of 211 articles were screened from the PubMed and Scopus databases. Eventually, after duplicate removal, record screening, and applying the snowball procedure, only 13 articles met the inclusion criteria and were selected for analysis [6,9,10,11,12,13,14,15,16,17,18,19,20]. These studies presented data on 25 patients. A flow diagram of the selection process is illustrated in Figure 1. Among the included cases, seven occurred in North and South America (53.8%), five in Europe (30.8%), one in Oceania (7.7%), and one in Asia (7.7%). Among the 13 included articles, 7 (53.8%) were case reports. Figure 2 represents the geographical distribution of all *Inquilinus* spp. infections worldwide. Table 1 shows the characteristics of the included studies in the present review.

### 3.2. Epidemiology of Inquilinus spp. Infections

The median age of patients with *Inquilinus* spp. infections was 19 years, varying from 2 to 60 years, while 60% (15 patients) were male. Regarding the patients’ medical history and predisposing risk factors, 24 out of 25 patients (96%) had pulmonary disease, with 23 (92%) having a history of cystic fibrosis and 1 patient (4%) having bronchiectasis and recurrent bronchitis, while 5 (20%) had received a lung transplant—all were patients with cystic fibrosis. The demographic and clinical characteristics of *Inquilinus* spp. cases are shown in Table 2.

### 3.3. Antimicrobial Resistance and Microbiology of Inquilinus spp. Infections

*Inquilinus* spp. were isolated from sputum cultures in 18 patients (72%), from bronchoalveolar fluid in 3 (12%), from blood cultures in 3 (12%), from lung explant culture in 1 (4%), and from pleural fluid, empyema rind, and thoracotomy wound tissue in 1 (4%). In three patients (12%), the method of isolation was not mentioned. *Inquilinus limosus* was the only identified species, and was identified in 21 patients (84%), while in the rest of the cases, *Inquilinus* spp. were mentioned as the identified pathogen. In 14 cases (56%), identification was performed with 16s rRNA sequencing. In six cases (24%), identification was performed with matrix-assisted laser desorption/ionization time-of-flight mass spectrometry (MALID-TOF MS). In six cases (24%), the means of pathogen identification was not mentioned. The antimicrobial resistance of *Inquilinus* spp. is shown in Table 3. In 17 out of 22 patients (77.3%), the infection was polymicrobial. More specifically, in 11 out of 17 patients with polymicrobial infection (64.7%), at least two other pathogens were isolated. The most commonly isolated pathogens were *Pseudomonas aeruginosa* in 14 patients (82.4%), *Aspergillus fumigatus* in 8 (47.1%), *Staphylococcus aureus* in 7 (41.2%), *Candida albicans* in 3 (17.6%), *Stenotrophomonas maltophilia* in 2 (11.8%), and non-tuberculous mycobacteria, *Paenibacillus amylolyticus*, and *Proteus mirabilis* in 1 (5.9%) each.

### 3.4. Clinical Presentation of Inquilinus spp. Infections

*Inquilinus* spp. infections most commonly involved the lower respiratory tract (in 24 patients; 96%) and the bloodstream (in 3 patients; 12%). Infective endocarditis was diagnosed in one patient (4%). The symptoms’ duration ranged from zero (acute onset) to more than three months.

### 3.5. Treatment and Outcome of Inquilinus Infections

Based on the available data, carbapenems were the most frequently administered antimicrobials (in 13 out of 19 patients with available data; 68.4%), followed by quinolones (in 11 patients; 57.9%) and aminoglycosides (in 10 patients; 52.6%). Of note, cephalosporins or other penicillins were rarely used, if ever. Surgical interventions were applied in combination with antimicrobial treatment in 2 out of 20 patients (10%). The median treatment duration for survivors was 28 days. The overall mortality rate was estimated at 4% (1 out of 25 patients), with the only death being directly associated with the *Inquilinus* spp. infection.

### 3.6. Lower Respiratory Tract Infection Due to Inquilinus Infection

Respiratory tract infection was diagnosed in 24 patients. The median age in this patient group was 18.5 years, and 15 patients were male (62.5%). Predisposing risk factors were lung disease in all patients. Lung transplantation had been performed in the past in five patients (20.8%). Among those with available data, 9.1% (2 out of 22 patients) also had bacteremia by *Inquilinus* spp. Most patients had polymicrobial infection, since 81% (17 out of 21 patients) also had other pathogens isolated from respiratory samples. Fever was present in 19% (4 out of 21), but no patient had sepsis. The median treatment duration was 22 days. Antimicrobial agents most commonly involved carbapenems, quinolones, and aminoglycosides. Overall mortality was 4.2% (1 out of 24 patients) and was specifically attributed to *Inquilinus* infection.

### 3.7. Bacteremia Due to Inquilinus

Bacteremia was diagnosed in three patients. Among them, one was male, and the median age was 38 years. Two of those patients had a lower respiratory tract infection due to *Inquilinus* spp. and were cystic fibrosis patients that had also undergone lung transplantation in the past. Only in one patient with an infection of the lower respiratory tract was the infection polymicrobial (*P. aeruginosa* and *A. fumigatus*). All patients had fever, but no patient had sepsis. One patient had infective endocarditis (without lower respiratory tract infection), and the clinical presentation also included heart failure. In this patient group, the median antimicrobial treatment was 42 days; carbapenems and aminoglycosides constituted the most widely used antimicrobials. No patient in this group died.

## 4. Discussion

This review presents the clinical and microbiological characteristics of infections by *Inquilinus* spp. in humans by evaluating all cases in the literature where such infections have been recorded. Two types of infections were documented: respiratory tract infection with or without bacteremia and infective endocarditis. All patients with a respiratory tract infection by *Inquilinus* spp. had a history of chronic respiratory disease, most commonly cystic fibrosis. Antimicrobial resistance to first-line beta-lactams was almost uniform, while quinolones and carbapenems retained significant antimicrobial activity against this pathogen and were very commonly used for treating such infections. Mortality from infections by *Inquilinus* spp. was very low.

Cystic fibrosis is a hereditary, chronic, progressive disease involving many systems, such as respiratory, gastrointestinal, and reproductive [21]. Due to mutations in the *CFTR* gene that affect the CFTR transporter that normally transports chloride and bicarbonate across the apical membrane of the secretory epithelia in the above-mentioned tissues, ion exchange is affected, leading to dysregulation of hydration of the tissues. For example, airway surface liquid hydration may be severely affected, leading to thick mucopurulent secretions in the airways that cannot be effectively cleared by the mucociliary system, leading to bacterial colonization and infection and to progressive structural damage of the lungs [2,21,22].

There is an association of cystic fibrosis with some specific microorganisms that tend to colonize patients’ airways and cause infections, while the microbiology of these infections is currently changing. Additionally, the increasing use of new technology that provides precise methods for pathogen identification, such as MALDI-TOF MS, leads to more accurate identification of even rare pathogens that may have been misidentified in the past [23,24,25,26]. For example, in a relatively recent study published in Chest, in which the microbiology of patients with cystic fibrosis reported in the Cystic Fibrosis Foundation Patient Registry from 2006 to 2012 was retrospectively evaluated, the epidemiology of pathogens was changing. The pathogens with the highest incidence were methicillin-sensitive *Staphylococcus aureus* (MSSA) and *Pseudomonas aeruginosa*, followed by methicillin-resistant *S. aureus* (MRSA) [27]. A significant decrease was noted during the evaluated period for *P. aeruginosa* strains and for the *Burkholderia cepacian* complex in terms of the annual percent change in overall prevalence and incidence, while a concomitant increase in the respective rates was noted for MRSA. At the same time, the prevalence of non-tuberculous mycobacteria and the *Mycobacterium avium* complex increased from 2010 to 2012 [27]. Other bacteria that are frequently isolated from patients with cystic fibrosis include *Haemophilus influenzae Stenotrophomonas maltophilia*, *Achromobacter xylosoxidans*, *Ralstonia* species, *Burkholderia gladioli*, *Cupriavidus* species, *Pandoraea* species, and *Inquilinus limosus* [24]. Other pathogens, such as fungi, have been also implicated in infections in patients with cystic fibrosis. Examples include *Aspergillus*, *Scedosporium*, *Candida* species, and *Exophiala dermatidis*. Moreover, viruses like the respiratory syncytial virus (RSV) and influenza virus are commonly involved in infections in such patients [24].

*Inquilinus* species was first isolated in 1999 and was further described in 2002 after taxonomic evaluation of bacterial isolates from the sputum of patients with cystic fibrosis [6,7]. The name of the single species, *Inquilinus limosus*, is derived from the Latin adjective for “slimy” or “full of slime”, due to the mucoid phenotype of this bacterium. Ever since, several reports of isolation of *I. limosus* from patients with cystic fibrosis have been published in Europe and North America [10,17,18,19,28,29]. The clinical significance of *I. limosus* isolation from patients with cystic fibrosis may not be always clear, since its isolation could denote colonization, not infection [30]. Additionally, some patients with cystic fibrosis may remain infected for prolonged periods of time [19,24,29]. The source of acquisition of this microorganism is unclear; however, scarce evidence suggests that the source could be nosocomial [9]. Additionally, there are scarce data regarding the interpatient spread of this microorganism. In a report of six patients with cystic fibrosis who were infected by *I. limosus*, no shared strains were detected, even among three of those patients who had been treated in the same cystic fibrosis clinic at the same time [17]. Watson et al. presented data of *I. limosus* infections in three co-habiting brothers suffering from cystic fibrosis, suggesting either inter-sibling transmission or acquisition from the same source [14]. In another study, *I. limosus* was identified as a commensal microorganism in the oral cavity of a cat, implying the possibility of zoonotic transmission [31].

*Inquilinus* infections are rare in clinical practice; however, they may be underreported. Their increasing identification in the last years may be associated with the increasing use of modern advanced technology for microbial identification. Indeed, in the vast majority of the studies included in the present review, pathogen identification was performed using 16s rRNA sequencing or MALDI-TOF MS. Indeed, these techniques have revolutionized microbiology, allowing the identification of rare microorganisms that would be very difficult to identify with the use of traditional morphological and biochemical characteristics of the pathogens [32,33,34]. Identifying *I. limosus* without these molecular methods and instead using routine diagnostics can be challenging [8]. *I. limosus* grows well on Chocolate and *Burkholderia cepacia*-selective agar, as well as on Columbia blood. Its growth can be slow, while its growth on MacConkey agar is not always successful [9,12,19,29,35]. The use of metabolic properties and biochemical reactions, as with the use of VITEK2 and API 20NE, is not appropriate for accurately identifying this microorganism since misidentification may occur. The most common misidentification of *Inquilinus* species involves *Roseomonas gilardii*, *Sphingomonas paucimobilis*, *Pseudomonas* species, or *Agrobacterium radiobacter* [11,12,19,29,30]. This misidentification could be associated with the fact that corresponding entries for *I. limosus* were not available in the databases of those identification methods [8,11,18,29]. Notably, even MALDI-TOF MS produced equivocal results in one study, yielding a borderline value, suggesting that identification was not definite and necessitating 16s rRNA sequencing to definitely identify the species causing the infection as *Inquilinus* [35].

Little is known about the pathogenesis of infections by *Inquilinus* species. In a relatively recent study investigating the role of emerging bacterial species in patients with cystic fibrosis, *Inquilinus* was found able to adapt and survive atmospheres with variable oxygen, as is the case in biofilms [36]. Moreover, there were no significant differences in the growth dynamics under anaerobic and microaerophilic conditions, suggesting that *Inquilinus* could survive under the conditions commonly encountered in biofilms. Given the mucoid morphology of the *Inquilinus* spp. strains and its resemblance to that of *P. aeruginosa* that also has a similar mucoid appearance and produces the exopolysaccharide (EPS) alginate, it is plausible to suppose that *Inquilinus* may also produce alginate. Alginate has been supposed to reduce the likelihood of phagocytosis, acting as a direct barrier and, thus, blocking opsonization [8,37]. In a study evaluating the possibility of EPS production by *I. limosus*, the bacterium was found to produce two mainly homopolymeric EPSs that had the same charge per sugar residue as alginate of *P. aeruginosa* [38]. This implies that the EPS produced by *I. limosus* could also act as a barrier against phagocytosis and opsonization. In another study evaluating the behavior of *I. limosus* in the presence of bronchial epithelial cells, *Inquilinus* was shown to invade the epithelial cells. Additionally, they were able to survive in the phagosomes, despite their acidic environment, even though their growth rate was slower [39]. Moreover, the epithelial cells that had been invaded by *I. limosus* could not induce the production of IL-6 and IL-8, suggesting a blunted pro-inflammatory response [39]. These findings suggest that *Inquilinus* species may chronically colonize the respiratory tract of people suffering from cystic fibrosis either through growth in biofilms or by invading the epithelial cells. Importantly, the *Inquilinus* bacterial cells that reside in biofilms have been found to be able to resist the antimicrobial activity of antibiotics, since the minimum concentrations needed to kill bacteria in the biofilm were much higher than the corresponding concentrations for the planktonic cells [40]. The mechanisms through which *Inquilinus* species have this adaptability are not yet clear; thus, future studies could shed light in these important pathogenetic mechanisms.

It can be difficult to differentiate between airway colonization and infection by *Inquilinus* spp. given the lack of published data on this particular issue. It is more likely that *I. limosus* airway colonization can present asymptomatically with patients that are clinically stable, while infections can lead to a range of clinical symptoms. It is not clear how or when a patient moves from a state of colonization to that of infection. Additionally, the pathogenetic mechanisms underlying this transition are also not known. Thus, little can be said regarding whether a particular patient is colonized or infected by *Inquilinus* spp. in patients with minimal symptoms or in patients with isolation of multiple microorganisms from cultures of respiratory specimens. Moreover, diagnostic tools that could be used to discriminate between colonization and infection are largely unknown, and, to date, clinical judgement is probably the only means to approach patients with *Inquilinus* spp. isolation in respiratory specimens. It is of note that there are studies supporting that even colonization of the respiratory tract of patients with cystic fibrosis by specific microorganisms can be associated with future respiratory function decline [41]. Whether this could also apply in the case of *Inquilinus* spp. is unknown. Thus, future studies should address the effect of colonization in patients with cystic fibrosis.

As shown in the studies included in the present review, many patients from whom *Inquilinus* was isolated suffered from polymicrobial infections. Isolation of *Pseudomonas*, *Aspergillus*, and *S. aureus* was common. This may be associated with underlying pathophysiological mechanisms involving the immune response. For example, a recent study showed that the presence of *P. aeruginosa* and *I. limosus* at the same time was associated with a significant increase in the production of neutrophil extracellular traps (NETs) compared to the presence of either of those two microorganisms alone [15]. This may be significant since, even though the presence of NETs is associated with increased microbial killing due to the microbial entrapment on these structures formed by extracellular DNA and proteolytic enzymes, their excessive production could increase sputum viscosity, thus allowing for increased colonization by microorganisms and progressive respiratory decline [42,43]. Thus, it is plausible to assume that *I. limosus*, along with other microorganisms colonizing or infecting the respiratory tract of a patient with cystic fibrosis, may lead to inflammatory response and the production of NETs, leading to a vicious cycle through bacterial persistence in the thicker secretions [8]. However, since there are no studies specifically evaluating the pathogenic mechanisms of *Inquilinus* colonization and infection, these speculations remain to be addressed in future studies.

Even though *I. limosus* has been mainly reported in patients with cystic fibrosis as a cause of respiratory tract infection, often associated with a decline in their respiratory function, other infections could potentially occur. This is underlined by one study where the patient suffered from bacteremia and infective endocarditis [9]. Notably, this patient did not have a history of cystic fibrosis and also developed an episode of infective endocarditis early after surgery, implying that the acquisition may have been nosocomial. This could imply that *Inquilinus* species could be, theoretically, implicated in other hospital-acquired infections. Yet, this remains to be evaluated in future studies involving a larger number of patients suffering from *Iqnuilinus* infections.

Previous studies have identified multiple chromosomally encoded multidrug resistance efflux pumps, four putative beta-lactamase genes, and seven penicillin-binding proteins from isolates of *I. limosus* that can partially explain the antimicrobial resistance patterns of these microorganism [44]. According to data extracted from the studies included in the present review, *Inquilinus* species had significant antimicrobial resistance to beta-lactams with the exception of carbapenems, as all strains with available data were resistant to aztreonam and the combination of piperacillin with tazobactam. Interestingly, antimicrobial resistance to quinolones was low, and resistance to aminoglycosides was near 50%; thus, these regimens could be used in infections by *Inquilinus* species if the results of antimicrobial resistance are known. However, empirical treatment with quinolones or carbapenems could be used, with the possibility of carbapenems being reserved for severe infections. Even though this review cannot provide strong recommendations, given the rarity of the evidence, appropriate empirical treatment is of paramount importance, especially in severe infections such as bacteremia, with significant rates of recurrence and mortality of the infection being noted when inadequate empirical treatment is provided [45]. However, given the lack of evidence and the inability for international scientific societies of infectious diseases to provide guidelines on the treatment of infections by these microorganisms, it is reasonable to consider carbapenems, quinolones, and aminoglycosides for treatment. Even though combination treatment is usually discouraged, even in patients with cystic fibrosis, as shown in a recent Cochrane review, in patients suffering severe infections [46], the role of combination treatment of *Inquilinus* spp. infections has not been evaluated. This is of particular note, since in some of the patients described in the studies included in the present review, more than one antimicrobial had been administered. To that end, it would be important to also have susceptibility testing with combinations of antimicrobials. This synergy testing would allow to better understand whether specific combinations of antimicrobials would be more appropriate for treating patients with severe infections [47].

Given the previously mentioned data on antimicrobial resistance, it is reasonable that carbapenems, aminoglycosides, and quinolones were the most commonly used antimicrobials for the treatment of *Inquilinus* infections. Notably, the treatment duration was quite long, which could be associated with a patient’s reduced or delayed clinical response that could be linked to the underlying cystic fibrosis. However, there are studies suggesting that a shorter duration of antimicrobials for patients with cystic fibrosis suffering an exacerbation of their pulmonary disease may be non-inferior to a longer duration [48]. This provides room for antimicrobial stewardship even in this population that may be at times difficult to treat.

The mortality of *Inquilinus* infections was minimal, with only one patient dying from the infection. Infections can be a significant cause of death in patients with cystic fibrosis, especially when lung transplantation has recently been performed [49]. However, the mortality rate of patients with cystic fibrosis and an infection varies depending on the severity and the type of the infection, as well as the rest of the patient’s medical history and overall status of health. For example, in a study involving children with cystic fibrosis admitted to a pediatric intensive care unit with acute respiratory failure due to pulmonary exacerbations, mortality was 17% at discharge, while a study with adult patients with cystic fibrosis who required mechanical ventilation showed a mortality rate of 44.5% during hospitalization [50,51]. However, chronic infections with specific pathogens such as *S. maltophilia* or a *B. cepacia* complex may be associated with significantly higher mortality rates. For example, chronic infection by *S. maltophilia* has been associated with a significantly higher risk of mortality or lung transplantation in patients with cystic fibrosis [52]. To that end, some patients in the studies that were included in the present study developed deterioration in their respiratory function after the diagnosis of the infection as a complication [14,17].

This study has some limitations. First of all, the literature search may not have included all relevant studies providing clinical and microbiologic characteristics, especially given the fact that differentiating between colonization and infection may not have been clear, even for the authors of the studies in some instances. The analysis in the present review was based solely on case reports and case series, which highly depend on accurate record-keeping. Additionally, some studies presented incomplete data, which restricted the scope of the analysis. Thus, this review only presents findings from studies that provided complete data, especially for mortality, basic demographic, and epidemiology. Moreover, this review identified only 13 studies with a small number of cases, which is a very small dataset to draw significant epidemiological or clinical conclusions. Thus, the small sample size and the potential for publication bias should be taken into account when reading this review. Finally, the exclusion of studies in languages other than English could introduce a potential sample bias; however, the number of such articles was minimal.

## 5. Conclusions

The present review provides important insights into the epidemiology, clinical presentation, antimicrobial resistance, and treatment and outcomes of *Inquilinus* infections, emphasizing key information regarding the pathogenic potential of this microorganism. *I. limosus* was the most commonly identified species, while almost all patients suffered a respiratory tract infection and had cystic fibrosis in their medical history. Polymicrobial infections were very common. *Inquilinus* was resistant to all beta-lactams with the exception of carbapenems, while antimicrobial resistance to quinolones was also low. These antimicrobials, along with aminoglycosides, were the most commonly used for the treatment of infections by this pathogen. Mortality was low. Due to the close association of *Inquilinus* with cystic fibrosis, *Inquilinus* should be considered in patients presenting with respiratory exacerbation, especially when treatment with simpler beta-lactams does not lead to clinical improvement. However, since microbial identification requires advanced molecular techniques such as MALDI-TOF MS or 16s rRNA sequencing, clinicians and laboratory professionals should be aware of this rare pathogen to allow adequate and timely diagnosis.

## Figures and Tables

**Table 1:** Characteristics of all included studies.

Author, Year	Number of Patients *	Gender	Age (Years)	Site of Infection (%)	Treatment (%)
Kiratisin et al., 2006 [9]	1	Female	38	Infective endocarditis, bacteremia	Carbapenem
Hayes et al., 2009 [10]	1	Male	20	LRTI	Quinolone, aminoglycoside
Goeman et al., 2015 [11]	1	Male	31	LRTI, bacteremia	Carbapenem, aminoglycoside
McHugh et al., 2016 [12]	1	Female	60	LRTI	Quinolone
Poore et al., 2016 [13]	1	Male	6	LRTI	Carbapenem, quinolone, aminoglycoside
Watson et al., 2021 [14]	3	3 Males	10, 13, and 22	LRTI	NR 3 (100)
Ríos-López et al., 2022 [15]	1	Male	12	LRTI	Quinolone
Farfour et al., 2023 [20]	3	1 Male 2 Females	22, 31, and 45	LRTI 3 (100), bacteremia 1 (33.3)	Cephalosporin 1 (33.3) NA 1 (33.3) Carbapenem 2 (66.7) Quinolone 2 (66.7) Aminoglycoside 2 (66.7)
Zobell et al., 2014 [16]	1	Female	13	LRTI	Carbapenem, aminoglycoside
Pitulle et al., 1999 [6]	1	Female	22	LRTI	NR
Chiron et al., 2005 [19]	3	1 Female 2 Males	10, 13, and 18	LRTI 3 (100)	Carbapenem 3 (100) Aminoglycoside 2 (66.7) Quinolone 2 (66.7)
Schmoldt et al., 2006 [17]	5	2 Males 3 Females	16, 17, 19, 20, and 35	LRTI 5 (100)	Carbapenem 4 (80) Quinolone 3 (60) Aminoglycoside 3 (60)
Bittar et al., 2008 [18]	3	3 Males	2, 15, and 21	LRTI 3 (100)	NR 3 (100)

LRTI: lower respiratory tract infection; NR: not reported; *: 25 patients were included in the present review and only one patient died.

**Table 2:** Characteristics of patients with *Inquilinus* species infection.

Characteristic	All Patients (*n* = 25) *	LRTI (*n* = 24) *	Bacteremia (*n* = 3) *
Age, years, median (IQR)	19 (13–26.5)	18.5 (13–22)	38 (31–45)
Male gender, *n* (%)	15 (60)	15 (62.5)	1 (33.3)
Predisposing factors			
Cystic fibrosis, *n* (%)	23 (92)	23 (95.8)	2 (66.7)
Lung transplantation, *n* (%)	5 (20)	5 (20.8)	2 (66.7)
Bronchiectasis and recurrent bronchitis, *n* (%)	1 (4)	1 (4.2)	0 (0)
Chronic heart disease, *n* (%)	1/19 (5.3)	0/18 (0)	1/2 (50)
Previous antimicrobial use within three months, *n* (%)	9/10 (90)	9/10 (90)	2/2 (100)
Lower respiratory tract infection, *n* (%)	24 (96)	24 (100)	2 (66.7)
Bacteremia, *n* (%)	3/23 (13)	2/22 (9.1)	3 (100)
Infective endocarditis, *n* (%)	1 (4)	0 (0)	1 (33.3)
Polymicrobial infection, *n* (%)	17/22 (77.3)	17/21 (81)	17/22 (77.3)
Clinical characteristics			
Fever, *n* (%)	5/22 (22.7)	4/21 (19)	3 (100)
Sepsis, *n* (%)	0/23 (0)	0/22 (0)	0 (0)
Treatment			
Carbapenem, *n* (%)	13/19 (68.4)	12/18 (66.7)	3 (100)
Quinolone, *n* (%)	11/19 (57.9)	11/18 (61.1)	1 (33.3)
Aminoglycoside, *n* (%)	10/19 (52.6)	10/18 (55.6)	2 (66.7)
Cephalosporin, *n* (%)	1/19 (5.3)	1/18 (5.6)	1 (33.3)
Surgical management, *n* (%)	2/20 (10)	2/19 (10.5)	1 (33.3)
Treatment duration, days, median (IQR)	28 (14–42)	22 (13–42)	42 (16–84)
Outcomes			
Deaths due to infection, *n* (%)	1 (4)	1 (4.2)	0 (0)
Deaths overall, *n* (%)	1 (4)	1 (4.2)	0 (0)

IQR: interquartile range; LRTI: lower respiratory tract infection; *: data are among the number of patients mentioned on top unless otherwise described.

**Table 3:** Antimicrobial resistance rates.

Antimicrobial Agent	Number of Patients *	Resistance (%)
Piperacillin/tazobactam	16/16	100
Aztreonam	14/14	100
Trimethoprim/sulfamethoxazole	6/7	85.7
Aminoglycosides	5/11	45.5
Quinolones	1/10	10
Carbapenems	1/16	6.3

*: Data show the number of strains being resistant to the respective antimicrobial divided by the number of strains with corresponding data. The rest of the cases did not have available data regarding antimicrobial resistance for the respective antimicrobial agent.

## Data Availability

No new data were created or analyzed in this study.
